# Validation of reference genes aiming accurate normalization of qPCR data in soybean upon nematode parasitism and insect attack

**DOI:** 10.1186/1756-0500-6-196

**Published:** 2013-05-13

**Authors:** Vívian de Jesus Miranda, Roberta Ramos Coelho, Antônio Américo Barbosa Viana, Osmundo Brilhante de Oliveira Neto, Regina Maria Dechechi Gomes Carneiro, Thales Lima Rocha, Maria Fatima Grossi de Sa, Rodrigo Rocha Fragoso

**Affiliations:** 1 Department of Cell Biology Graduate Program in Molecular Biology, University of Brasília, Brasília, DF, Brazil; 2 Embrapa Genetic Resources and Biotechnology, Laboratory of Molecular Plant-Pest Interaction, PqEB Final Av. W/5 Norte, Brasília, DF, Brazil; 3 Catholic University of Brasília, Graduate Program in Genomic Sciences and Biotechnology, Brasília, DF, Brazil; 4 Faculdades Integradas do Planalto Central – Faciplac, Brasília, DF, Brazil; 5 Embrapa Cerrados, Laboratory of Phytopathology, Planaltina, DF, Brazil

**Keywords:** *Glycine max*, *Meloidogyne incognita*, *Anticarsia gemmatalis*, Gene expression, Real-time PCR

## Abstract

**Background:**

Soybean pathogens and pests reduce grain production worldwide. Biotic interaction cause extensive changes in plant gene expression profile and the data produced by functional genomics studies need validation, usually done by quantitative PCR. Nevertheless, this technique relies on accurate normalization which, in turn, depends upon the proper selection of stable reference genes for each experimental condition. To date, only a few studies were performed to validate reference genes in soybean subjected to biotic stress. Here, we report reference genes validation in soybean during root-knot nematode (*Meloidogyne incognita*) parasitism and velvetbean caterpillar (*Anticarsia gemmatalis*) attack.

**Findings:**

The expression stability of nine classical reference genes (*GmCYP2*, *GmELF1A*, *GmELF1B*, *GmACT11*, *GmTUB*, *GmTUA5*, *GmG6PD*, *GmUBC2* and *GmUBC4*) was evaluated using twenty-four experimental samples including different organs, developmental stages, roots infected with *M. incognita* and leaves attacked by *A. gemmatalis*. Two different algorithms (*geNorm* and *NormFinder*) were used to determine expression stability. *GmCYP2* and *GmUBC4* are the most stable in different organs. Considering the developmental stages, *GmELF1A* and *GmELF1B* genes are the most stable. For spatial and temporal gene expression studies, normalization may be performed using *GmUBC4*, *GmUBC2*, *GmCYP2* and *GmACT11* as reference genes. Our data indicate that both *GmELF1A* and *GmTUA5* are the most stable reference genes for data normalization obtained from soybean roots infected with *M. incognita*, and *GmCYP2* and *GmELF1A* are the most stable in soybean leaves infested with *A. gemmatalis*.

**Conclusions:**

Future expression studies using nematode infection and caterpilar infestation in soybean plant may utilize the reference gene sets reported here.

## Background

Soybean is a crop of enormous economic importance due to several nutritional and industrial applications. The soy grain is the world's leading source of protein and vegetable oil [1]. Nutritional benefits are due to high levels of essential amino acids and fatty acids, vitamins and minerals [2]. In addition to the extensive use of the grain in the food industry (animal and human foodstock), the soybean is also used in the production of biodiesel [3].

However, despite the great expansion of soybean acreage, insect-pests and diseases have reduced the crop productivity [4]. *Anticarsia gemmatalis*, known as the velvetbean caterpillar, attacks the leaves causing severe plant damage. This caterpillar is native from tropical and subtropical areas of the western hemisphere and is commonly found in tropical America, being a major pest of soybean crops in Brazil, a major producer of the grain [5]. They are able to feed on young leaves, causing reduction of leaf area and photosynthetic rate. When in large populations, the damages are so severe as the complete loss of leaves, including the ribs and the petiole, which causes up to 100% of production losses [6].

The root-knot nematode *Meloidogyne incognita* is probably the most important nematode in agriculture due to its worldwide distribution and wide variety of host plants [7,8], being widely distributed in soybean crops, causing an average of 5% of crop losses around the world [9]. The infective stage, known as second-juvenile (J2), invades the root tips and migrates in root tissues between cell walls to reach the vascular cylinder, where it secretes proteins from esophageal glands that induce giant cells formation resulting in a structure named feeding site. Hyperplasia and hypertrophy of cortical cells are achieved by interfering with plant gene expression, what therefore leads to gall formation [10].

Aiming to understand the plant-pest interactions, several functional genomics studies have been done [11]. The large-scale technique of gene expression profiling usually reported is the use of microarrays, some of them initiated by laser capture microdissection (LCM) at giant cells [12,13]. However, these results demand a validation step to confirm differential gene expression, which is made by quantitative PCR. qPCR is currently the most accurate technique to quantify transcript expression, due to its high sensitivity, reproducibility, high resolution, wide dynamic range, and no post-PCR processing [14].

The qPCR reliability, however, depends on normalization, to correct for non-biological variations such as sample quantity and quality, RNA preparation, cDNA synthesis and sample dilution and pipetting errors [15]. The commonly used reference genes in plant are related with basal cell metabolism (housekeeping genes), these being structural genes of the cytoskeleton (actin and tubulin), genes involved in protein folding (cyclophilin and metalloproteases), genes involved in protein degradation (ubiquitin), in protein synthesis (elongation factor) and glucose metabolism (glyceraldeide-3-phosphate dehydrogenase, glucose-6-phosphate dehydrogenase) [15-17]. All these genes are referred to as constitutive genes, however, several studies have demonstrated that levels of transcripts of these genes may vary considerably under different experimental conditions, tissues and life cycle [16]. Therefore, there is a demand for stable reference genes aiming their use in different experimental settings.

In this work, the expression stability of nine reference genes was analyzed in various organs, at different developmental stages of soybean and during leaf infestation with velvetbean caterpillar *A. gemmatalis* and root infection with the root-knot nematode *M. incognita*.

## Methods

### Plant material

The BRSGO Raissa soybean plants were grown at 25 ± 4°C in a greenhouse. Samples were collected at three soybean developmental stages: Vegetative 4 (V4 - characterized by the presence of the third fully developed trifoliate leaf), Reproductive 2 (R2 - full flowering) and Reproductive 4 (R4 - fully developed pods). Plant organs (root, stem, leaf, flower and pod) were collected and pooled (Additional file 1).

### Soybean roots inoculation with *Meloidogyne incognita*

Santa Rosa soybean plants were grown in acclimatized chamber (25–28°C, 70% humidity and 16 h photoperiod). Raissa soybean variety was not used to the nematode interaction study because it shows natural resistance to *Meloidogyne incognita*. The nematodes previously isolated from soybean fields, were multiplied in tomato plants for 35 days. After this period, the roots were collected, ground in a blender with 0.5% (v/v) sodium hypochlorite and the material were separated in 100 and 500 mesh sieves. Eggs obtained in 500 mesh sieve was mixed with kaolin and centrifuged at 2500 g for 10 minutes. The precipitate was resuspended in 50% sucrose and centrifuged at 2500 g for 1 min. The suspension of eggs free of impurities was collected from the supernatant in 500 mesh sieve and placed in the hatching chamber at 28°C for 48 hours. Juveniles (J2) were then collected and counted in a Peters chamber. Seedlings at VC (vegetative cotiledonar) on soil pots were inoculated at four points around the stem with approximately 5,000 *M. incognita* J2. The root tips, galls and non-inoculated control were collected at 7, 14, 21, 28 DAI (Additional file 1). Additional roots were collected and stained with acid fuchsin at each time point [18] to monitor nematode infection (Additional file 2).

### Soybean infestation with the velvetbean caterpillar (*Anticarsia gemmatalis*)

The BRSGO Raissa soybean plants were grown in acclimatized chamber as described in the previous section. Soybean leaves at the V4 stage (the phase often attacked by defoliating caterpillars) were subjected to caterpillars of fourth-instar *A. gemmatalis* obtained from rearing on artificial diet. A total of 25 caterpillars were distributed in two trifoliate leaves of the same plant to start the feeding process. The leaves from three plants were then collected at 15, 30, 60 and 180 minutes after caterpillar wounding (Additional file 1). As a control, soybean leaves without any contact with the caterpillars were also collected (Additional file 3).

### Extraction of total RNA and cDNA synthesis

In all treatments, samples were collected from 3–5 plants and pooled, frozen in liquid nitrogen and stored at −80°C for further RNA extraction. All procedures were repeated in a distinct setting in order to obtain a biological replicate. Total RNA was extracted using Trizol reagent (Invitrogen, CA, USA) according to the manufacturer's protocol. RNA quantification was performed using the ND-1000 spectrophotometer NanoDrop. The integrity of total RNA was analyzed by 260/280 nm ratio and confirmed by electrophoresis (Additional file 4). Before cDNA synthesis, RNA was treated with DNase I (Amplification Grade DNase kit - Invitrogen) according to the manufacturer's instructions to eliminate any possible contamination with genomic DNA. cDNA was synthesized from 1 μg of total RNA using the kit SuperScript™ III First-Strand Synthesis Supermix for qRT-PCR (Invitrogen) according to the manufacturer's instructions. The cDNA samples were stored at - 20°C until needed.

### PCR primers design

Primers were designed using the Primer 3 software and checked for the presence of hetero and homodimers using OligoTech 1.00. Six pairs of primers were designed to align in different exons as a strategy to identify the presence of contaminant genomic DNA in the cDNA samples (Table 1).

**Table 1:** Primer sequences and amplicon characteristics of tested genes

**Gene symbol**	**Forward primer sequence (5’- 3’)**	**Reverse primer sequence (5’- 3’)**	**Amplicon length (bp)**	Primer location*	**Efficiency (%)**
*GmCYP2*	CGGGACCAGTGTGCTTCTTCA	CCCCTCCACTACAAAGGCTCG	154	S	98,3
*GmELF1A*	GACCTTCTTCGTTTCTCGCA	CGAACCTCTCAATCACACGC	195	D	102,4
*GmTUA5*	AGGTCGGAAACTCCTGCTGG	AAGGTGTTGAAGGCGTCGTG	159	S	101,7
*GmELF1B*	GTTGAAAAGCCAGGGGACA	TCTTACCCCTTGAGCGTGG	118	D	92,5
*GmACT11*	CGGTGGTTCTATCTTGGCATC	GTCTTTCGCTTCAATAACCCTA	142	D	104,1
*GmUBC2*	TCCCCTCACACCCTTCCTC	CCATCCCAAGGGGTGTCAT	155	D	107,6
*GmTUB*	CCTCGTTCGAATTCGCTTTTTG	CAACTGTCTTGTCGCTTGGCAT	161	S	96,4
*GmG6PD*	ACTCCTTGATACCGTTGTCCAT	GTTTGTTATCCGCCTACAGCCT	126	D	110,7
*GmUBC4*	GAGCGAGCAGTTTCAGAC	CATAGGAGGGACGATACG	168	D	98
*GmRB7*	TTGTAGGTGTCTCCGTCGC	AATGCTCTTGGCGGTGATG	179	S	87,3

*Single exon (S) and different exons (D).

### qPCR and data analyses

The quantitative real-time PCR amplifications were performed using the Mastercycler Realplex (Eppendorf) thermal cycler. Rox plus Sybr Green Master Mix 2X (LGC) were used with 200 nM of each primer (sense and antisense) and 2 μL of cDNA (40-fold dilution) for each experimental condition. All experiments were performed in experimental triplicate and biological duplicate. The PCR cycling conditions were: 95°C for 15 min to activate the hot-start Taq DNA polymerase, 40 cycles at 95°C for 20 s, 55°C for 20 s and 72°C for 20 in soil pots. The raw data of fluorescence for all runs were imported into the *Real-time PCR Miner* software [19] in order to determine the Ct value and the PCR efficiency. The analyses of *GmRB7* expression were performed using *qBase*^*Plus*^ software [20].

### Analysis of reference genes expression stability

The Ct values relative to both biological replicates were imported into *qBase v.1.3.5* and the arithmetic mean of the Ct value was calculated and submitted to the *NormFinder* software to rank the most suitable reference genes. This software ranks the genes according to their stability of expression in a set of experimental conditions, and selects the most stable combination of two genes to establish the normalization factor, based on the lowest intra-and inter-group variation [21].

The same procedure was performed for analysis in *geNorm*^*PLUS*^. Moreover, Ct values were imported into *qBase*^*PLUS*^ software, which combines the calculation of relative quantities with *geNorm* analysis in a single software. The *geNorm*^*PLUS*^ software determines the most stable reference gene based on the M value, which means that genes with low M value have a high expression stability. This value is based on the geometric mean of genes and on the average pairwise variation of gene against all others in the different samples. The algorithm also calculates the pairwise variation (V_n_/V_n+1_) between two factors standards (FN_n_/FN_n+1_) to determine how many genes are required for accurate normalization and the combined M value to the more stable genes. A cut-off point of 0.15 was established, in which the inclusion of an additional gene has no effect on the pairwise variation.

## Findings

Some reference genes have been previously validated in soybean using different organs, at different life stages, light exposition treatments and during infection with the Asian soybean rust (*Phakopsora pachyrhizi*) [15-17]. There is a lack of validated reference genes, however, during nematode parasitism and caterpillar wounding.

The nine commonly used reference genes evaluated here were: *GmCYP2*, *GmELF1A*, *GmELF1B*, *GmACT11*, *GmTUB*, *GmTUA5*, *GmG6PD*, *GmUBC2* and *GmUBC4*[15] (Additional file 5). The specificity of each gene amplification was evaluated using the dissociation curve (Additional file 6). Except for the *GmTUB*, all amplifications resulted in just one peak. All reference gene candidates showed similar ranges of cycle of threshold (Ct) from 21.31 (*GmCYP2*) to 29.46 (*GmTUB*) (Additional file 7). The amplification efficiencies were determined individually to each well by the *Miner* software and were above 90% for almost all genes in every treatment, except for some genes, especially *GmRB7* (Table 1) in root gall samples (Additional file 7).

**Figure 1:**
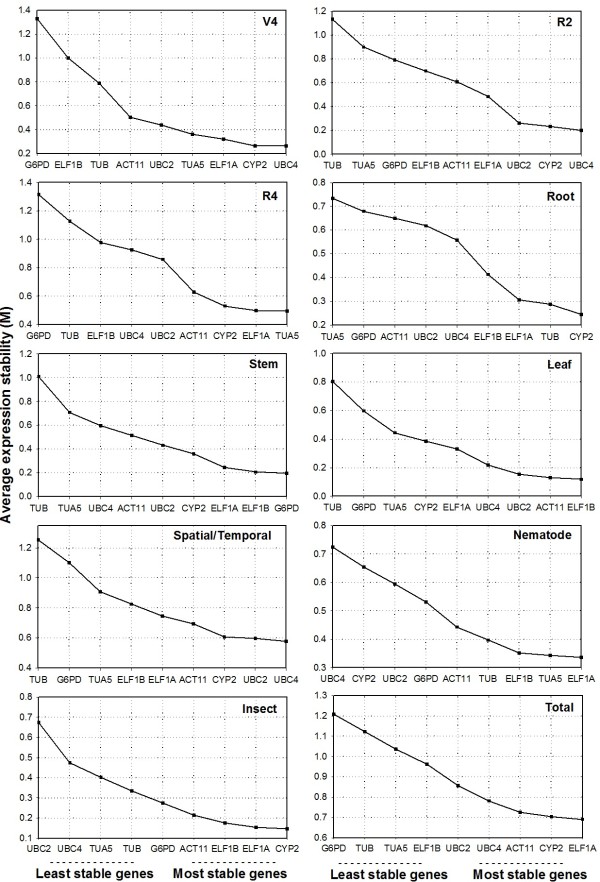
**Expression stability values (M) and ranking of the candidate reference genes as predicted by *geNorm*.** Average expression stability values (M) were measured using stepwise exclusion of the least stable gene to organize candidate genes from the least (left) to the most stable (right). Different organs at three developmental stages (V4, R2 and R4). Developmental series in different plant organs (Root, Stem and Leaf). All organs and developmental stages together (Spatial/Temporal). Biotic stress treatments: Nematode-infected root (Nematode) and leaf infested with caterpillar (Insect). All conditions combined (Total).

The validation of reference genes in soybean was determined according to *geNorm*, which demonstrated that *GmCYP2* and *GmUBC4* gene expression were the most stable amongst different organs at V4 and R2 stages (Figure 1), with a combined M value for both genes of 0.093 at V4 stage and of 0.149 at R2 stage. Jian and colleagues [15] obtained similar results with CYP2 as the second most stable gene to be used in normalization in different organs of soybean. On the other hand, Ruibo Ru and collaborators [16] observed that CYP2 showed a medium stability profile in different organs, when compared to other candidate genes, and that CYP2 was the least stable among different developmental stages in soybean. At soybean R4 stage, the most stable genes are *GmTUA5* and *GmELF1A* (Figure 1) with a combined M of 0.401.

In the developmental series, *GmTUB* and *GmCYP2* are the most stable genes in roots (Figure 1), showing a combined M of 0.235, whereas *GmG6PD* and *GmELF1B* in stem (M = 0.094), and *GmELF1B* and *GmACT11* in leaves (M = 0.055). Jian and colleagues [15] suggested ELF1B as the most stable gene in all samples for soybean expression analyses, whereas we detected *GmELF1A* as the most stable, reinforcing the well established concept that translation is a highly stable process. In all tested experimental conditions, the *GmELF1A* gene was the most stable and, on the other hand, *GmG6PD* was the most variable (Figure 1). Considering spatial and temporal gene expression together, four genes are required for accurate normalization: *GmUBC4*, *GmUBC2*, *GmCYP2* and *GmACT11* with a combined M value of 0.693.

Gene expression studies in plants subjected to pathogens and pests attack have increased the knowledge in plant defense mechanisms, what could be applied in biotechnological strategies to improve pathogen and pest control [22]. Biotic stresses cause extensive changes in plant gene expression [10,23], what hinders data normalization studies. Some previous studies reported that several housekeeping genes, usually used as reference genes, demonstrated expression variation during biotic stress in plants [17,24,25]. Previous microarray analyses carried out in soybean inoculated with *M. incognita* at 12 and 10 DAI have revealed that *M. incognita* not only activates responses of plant defense but also induces morphological and physiological changes in roots during feeding site establishment and maintenance [13]. Indeed, Ibrahim et al. [13] observed expression changes greater than 1.5-fold in 1,867 genes involved in cell division, cell wall remodeling, carbon and energy metabolism, defense-related genes and transcriptional factors, due to the extensive morphological changes in plant cells upon nematode infection. Therefore, some housekeeping genes associated with cell division, cytoskeletal structure and glycolytic pathway, commonly used as reference genes, are not suitable for this purpose due to its demonstrated up-regulation in infected roots, as a response to biotic stress [13].

In this report, we validate the most stable genes in galls of soybean inoculated with the root-knot nematode *M. incognita*. The most stable genes here described are *GmELF1A* and *GmTUA5* (M = 0.316), considering the eight samples of non-inoculated and inoculated roots at four points of the time course, although *GmUBC4* was the most variable (Figure 1). A similar work to identify reference genes was performed on *A. thaliana* inoculated with *M. incognita* and *Heterodera schachii*, in which roots were collected at 5, 10 and 15 DAI [6]. It was verified that *ELF1A* was up-regulated in galls as well as in syncytia, demonstrating that this gene is not suitable for normalization of expression studies. In potato, ELF1A presented a highly stable expression pattern in plants submitted to biotic (the late blight caused by *Phytophthora infestans*) and abiotic (cold and salt) stresses [24].

*GmUBC4* and *GmUBC2* genes have not shown a stable expression pattern in galls in our study. This low stability observed is in accordance with previous studies, which have shown that UBCs are modulated in nematode feeding sites [10]. The *GmCYP2* also showed a wide variation in root galls. Some studies have reported differential induction of cyclophilin during development or exposure to certain stresses [26]. Conditions such as exposure to mercuric chloride [27], heat shock, virus infection, the growth regulators ethephon and salicylic acid [28] have been shown to induce the expression of CYP in plants.

In this work, the *GmACT11* gene did not show a highly stable expression pattern in root galls. Indeed, Hoffman and Grundler [6] reported that Actin2 expression varied considerably in roots infected with *H. schantii* and with *M. incognita*, being down-regulated upon infection and its progression. Almeida Engler et al. [29] reported cytoskeleton changes in the syncytia and galls at nematode feeding sites, via actin and tubulin depolymerization. The actin gene showed variable expression also in potato plants exposed to the late blight, salt stress and cold stress, suggesting that actin is not suitable as a normalization reference in conditions of abiotic and biotic stresses [24].

In leaves attacked by the soybean caterpillar, we observed that *GmCYP2* and *GmELF1A* genes were the most stable, with a combined M value of 0.092 (Figure 1), considering all the five samples. A high stability of ELF-4A1 expression was also previously observed in microarray experiments on *A. thaliana* plants infested with *Pieris rapae*, *Frankliniella occidentalis* and *Myzus persicae*[30]. In that same report it was also demonstrated a higher expression stability of *Tubulin β-4*, *actin 2*, *aquaporin PIP-1B* and 40S ribosomal protein S16 genes, suggesting that these genes are suitable candidates for normalization upon infestation with caterpillars, thrips and aphids [30]. Rehrig et al. [25] analyzed twelve traditional reference genes in *A. thaliana* subjected to the attack by two caterpillars, *Spodoptera exigua* and *Pieris rapae* and reported that all analyzed reference genes are not stable after the attack of these insects. The authors suggested a method of normalization using mRNA quantitation in combination with the addition of an external mRNA (luciferase mRNA), commercially available as the normalization factor in studies involving herbivores [25]. We confirmed here that expression of the actin gene is among the most stable after *A. gemmatalis* larvae attack using both *geNorm* and *NormFinder* softwares (Figure 1, Table 2). Rayapuram and Baldwin [31] indicated that actin expression is not affected in plants of *Nicotiana attenuata* after *Manduca sexta* infestation.

**Table 2:** **Expression stability values and rankings of the reference genes calculated by *NormFinder *software**

**A**									
**Organs V4**		**Organs R2**		**Organs R4**		**Root - development**		**Stem - development**	
Ranking	Stabilityvalue	Ranking	Stability value	Ranking	Stability value	Ranking	Stability value	Ranking	Stability value
*GmELF1A*	0,189	*GmACT11*	0,187	*GmCYP2*	0,169	*GmELF1A*	0,189	*GmCYP2*	0,335
*GmUBC2*	0,321	*GmTUA5*	0,451	*GmELF1A*	0,180	*GmUBC2*	0,190	*GmTUA5*	0,386
*GmACT11*	0,429	*GmELF1A*	0,499	*GmACT11*	0,188	*GmCYP2*	0,306	*GmUBC2*	0,430
*GmUBC4*	0,535	*GmG6PD*	0,549	*GmTUA5*	0,217	*GmUBC4*	0,331	*GmACT11*	0,490
*GmTUA5*	0,593	*GmUBC4*	0,617	*GmUBC4*	0,385	*GmTUA5*	0,390	*GmELF1A*	0,672
*GmELF1B*	0,605	*GmELF1B*	0,692	*GmELF1B*	0,396	*GmELF1B*	0,557	*GmG6PD*	0,767
*GmG6PD*	0,896	*GmUBC2*	0,703	*GmUBC2*	0,450	*GmG6PD*	0,742	*GmUBC4*	0,792
*GmCYP2*	1,046	*GmCYP2*	0,738	*GmG6PD*	0,980	*GmACT11*	0,899	*GmELF1B*	0,932
*GmTUB*	2,966	*GmTUB*	4,138	*GmTUB*	2,095	*GmTUB*	1,393	*GmTUB*	3,529
Best combination	Stability value	Best combination	Stability value	Best combination	Stability value	Best combination	Stability value	Best combination	Stability value
*GmELF1A*	0,203	*GmACT11*	0,250	*GmCYP2*	0,130	*GmELF1A*	0,167	*GmCYP2*	0,259
and *GmUBC2*		and *GmTUA5*		and *GmELF1A*		and *GmUBC2*		and *GmTUA5*	
**B**									
**Leaf - development**		**Spatial/Temporal**		***M. incognita*-inoculated root**		***A. gemmatalis*-infested leaf**		**Total**	
Ranking	Stability value	Ranking	Stability value	Ranking	Stability value	Ranking	Stability value	Ranking	Stability value
*GmELF1A*	0,183	*GmACT11*	0,298	*GmELF1A*	0,1333	*GmCYP2*	0,060	*GmACT11*	0,089
*GmACT11*	0,230	*GmTUA5*	0,349	*GmACT11*	0,1660	*GmELF1A*	0,067	*GmELF1A*	0,124
*GmUBC2*	0,412	*GmUBC2*	0,448	*GmTUA5*	0,2221	*GmACT11*	0,081	*GmTUA5*	0,236
*GmCYP2*	0,433	*GmELF1A*	0,493	*GmG6PD*	0,3143	*GmG6PD*	0,087	*GmG6PD*	0,348
*GmELF1B*	0,451	*GmG6PD*	0,527	*GmUBC2*	0,3177	*GmELF1B*	0,121	*GmELF1B*	0,425
*GmTUA5*	0,545	*GmCYP2*	0,669	*GmTUB*	0,3267	*GmTUA5*	0,145	*GmCYP2*	0,559
*GmUBC4*	0,670	*GmELF1B*	0,722	*GmCYP2*	0,3951	*GmUBC4*	0,222	*GmUBC4*	0,621
*GmG6PD*	0,925	*GmUBC4*	0,748	*GmELF1B*	0,4186	*GmTUB*	0,293	*GmUBC2*	0,647
*GmTUB*	1,219	*GmTUB*	2,638	*GmUBC4*	0,7903	*GmUBC2*	0,948	*GmTUB*	1,246
Best combination	Stability value	Best combination	Stability value	Best combination	Stability value	Best combination	Stability value	Best combination	Stability value
*GmELF1B* and GmTUA5	0,177	*GmACT11* and GmTUA5	0,176	*GmACT11* and GmELF1A	0,133	*GmCYP2* and GmELF1A	0,057	*GmACT11* and GmELF1A	0,084

Stability values are listed from the most stable genes to the least stable.

**Figure 2:**
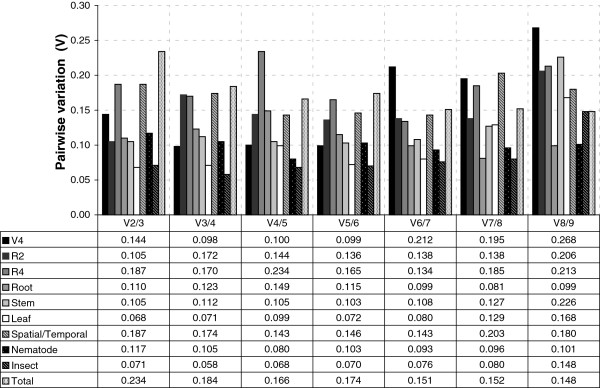
**Pairwise variation (V) analysis of the candidate reference genes as predicted by *geNorm*.** The Pairwise variation (V_n_/V_n+1_) was analyzed using the normalization factors NF_n_ and NF_n+1_ to determine the optimal number of reference genes required for effective normalization of qPCR data.

The optimal number of reference genes for normalization was calculated by the *geNorm* software. According to *geNorm*, two genes are required to normalize the target gene in different organs between V4 (V = 0.144) and R2 (V = 0.105), due to the V value below the cut-off value (0.15) suggested by Vandesompele et al. [32], excluding the need to add another gene to form the normalization factor (Figure 2). Among the different organs in the R4 stage, *geNorm* recommended six genes to form the normalization factor (V = 0.134), however, a low combined M value (0.401) was detected when only *GmTUA5* and *GmELF1A* are used. In the series of development for all organs (root, stem and leaf), only the two most stable genes are required for normalization. Considering all organs at all stages, four genes are required for normalization (V = 0.143). In the two stress treatments (nematod infection V = 0.117 and velvetbean caterpillar wounding/feeding V = 0.071), only two genes are required for normalization, indicating low variation of reference genes stability in these samples.

According to *NormFinder*, the most stable gene amongst organs at V4 stage was *GmELF1A*, with a 0.189 stability value, and the best combination of two genes to form the normalization factor genes were *GmELF1A* and *GmUBC2*, with a stability value of 0.203 (Table 2). At R2 stage, *GmACT11* was the most stable gene and *GmACT11* and *GmTUA5* were the most stable gene set to form the normalization factor with a stability value of 0.250. At R4 stage, the most stable genes were *GmCYP2*, *GmELF1A*, *GmACT11* and *GmTUA5*, which is similar to the results obtained with the *geNorm* software (Figure 1), However, the most stable genes for the normalization were *GmCYP2* and *GmELF1A* with a 0.130 stability value. In the development series, the gene *GmELF1A* showed high stability in the three organs analyzed, but the most stable in stem was the *GmCYP2* gene, with a 0.335 stability value, and the *GmELF1A* was the most stable gene in root and leaf (Table 2).

The classification generated by *NormFinder* was slighty distinct from that determined by the *geNorm* software, which can be explained by intrinsic differences in the mathematical models applied in each software. *geNorm* determines the most stable reference genes from a set of genes and a given panel of cDNA samples. It computes the best combination of reference genes to compose the normalization factor based on the geometric mean of the genes and the average pairwise variation [32]. The *NormFinder* identifies the best combination of two genes to form the normalization factor among a set of candidates. It performs a model-based variance calculation that estimates the variation of intra and intergroup expression and calculates the stability expression value of each gene [21]. This model selects the best combination of genes with the best normalization factor, i.e., with less variation of intra and intergroup, whereas models based on pair-wise variation, like *geNorm*, selects genes with lower variation intragroups and with the same variation intergroups [21,32].

**Figure 3:**
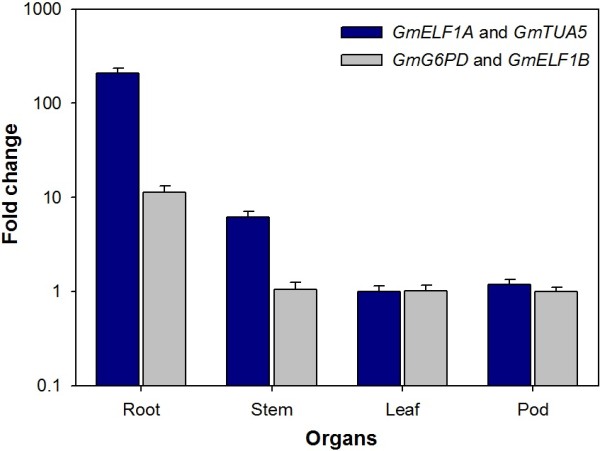
**Relative quantification of *GmRB7 *expression in different organs.** The root-specific aquaporin *GmRB7* transcript quantification was determined in different plant organs at the R4 stage normalized with the most stable pair of reference genes *GmELF1A* and *GmTUA5* (Blue) and with the least stable pair of reference genes *GmG6PD* and *GmELF1B* (Gray), determined by *geNorm*. *GmRB7* expression in different organs was calculated relative to leaf. The Y-axis represents relative expression values in fold change. The bars represent standard deviations.

The aquaporin *GmRB7* transcript abundance pattern in different organs at R4 stage was confirmed using the most or the least stable gene pairs for normalization (Figure 3). The *GmRB7* gene encodes for a multipass transmembrane water transporter protein, known to be abundant in root tissues. *GmRB7* was chosen in this study because it was previously characterized to be a root-specific gene whose expression is induced by root-knot nematodes at giant cells of feeding sites [33]. The *RB7* gene transcripts, first analyzed in tobacco plants by *in situ* hybridization was localized in the root meristem and immature central cylinder regions [33].

**Figure 4:**
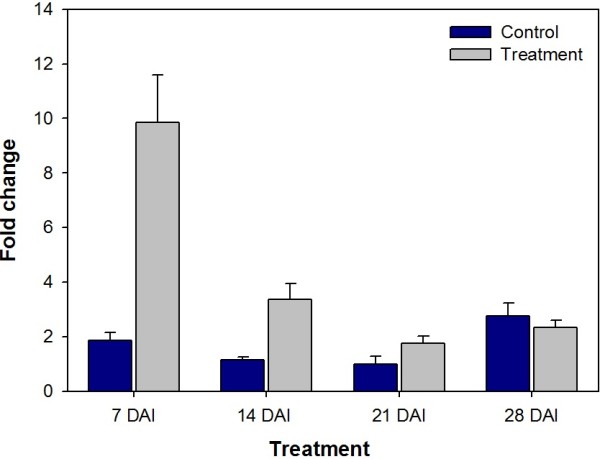
**Relative quantification of *GmRB7 *expression in soybean roots infected with *M. incognita*.** Abundance of *GmRB7* transcript was determined relatively to non-infected roots during the four-week experimentation period and normalized with *GmTUA5* and *GmELF1A*. The four time points are shown at the X-axis, whereas samples of non-inoculated roots are in blue bars and samples of inoculated roots in gray. The bars represent standard deviations.

When *GmRB7* gene expression was normalized using the two most stable genes (*GmELF1A* and *GmTUA5*) according to *geNorm*, the root-specific expression pattern was 208-fold higher than leaf (Figure 3). When the normalization was performed using the two least stable genes (*GmG6PD* and *GmELF1B*) the root expression was only 11-fold higher than leaf (Figure 3). Thus, normalization using reference genes with low stability can mask tissue-specificity and/or be innacurate. The pattern of aquaporin expression was also analyzed in roots inoculated with root-knot nematodes, once its expression is nematode-induced [33]. It was found that *GmRB7* expression increased 9.86- and 3.37-fold at 7 and 14 DAI, respectively (Figure 4).

## Conclusion

In conclusion, the validation of reference genes in soybean hereby presented demonstrates that *GmELF1A* and *GmTUA5* are the most stable genes during the infection of roots by *M. incognita* and *GmCYP2* and *GmELF1A* are the most stable genes during *A. gemmatalis* leaf attack. The reference genes validated in this work enables more accurate and reliable normalization of qPCR results for gene expression studies in soybean during interaction with the root-knot nematode and the velvetbean caterpillar.

## Abbreviations

GmCYP2: Cyclophilin 2; GmELF1A: Translation elongation factor 1α; GmELF1B: Translation elongation factor 1β; GmACT11: Actin 11; GmTUB: β-tubulin 5; GmTUA5: α-tubulin; GmG6PD: Glucose-6-phosphate dehydrogenase; GmUBC2: Ubiquitin-conjugating enzyme E2, UBC2 family member; GmUBC4: Ubiquitin-conjugating enzyme E2, UBC4 family member; GmRB7: Aquaporin; qPCR: quantitative real-time polymerase chain reaction; DAI: Day after inoculation; Ct: Cycle threshold; cDNA: Complementary DNA.

## Competing interests

The authors declare that they have no competing interests.

## Authors’ contributions

VJM was responsible for conducting the experiments, RNA and cDNA samples preparation, qPCR experiments and drafting the manuscript. RRC participated in experimental design and *A. gemmatalis* assays. AABV and TLR participated in experimental design and manuscript writing. OBON aided in the conduction and preparation of qPCR runs. RMDGC participated in the design of experimental assays with nematodes and assisted in the collection of root galls. MFGS participated in the supervision of the work and in experimental design. RRF participated in experimental design and supervision of the study, data analysis and manuscript writing. All authors read and approved the final manuscript.

## Supplementary Material

Additional file 1 Set of samples (organ/treatment) used for gene expression analysis. Click here for file

Additional file 2 **Progress of soybean root infection with *M. incognita *revealed by acid fuchsin staining. (A) 7 DAI, second-stage juvenile (J2) during penetration and migration into root; (B) 14 DAI, gall formation by J2-J3 in the vascular cylinder; (C) 21 DAI, root knot completely developed; (D) 28 DAI, adult female during egg posture and egg mass.** Click here for file

Additional file 3 **Progress of soybean leaf infestation with *A. gemmatalis*. Twenty-five larvae of fourth instar of *A. gemmatalis *were transferred to each soybean trifolium. Leaves were collected at (A) 15, (B) 30, (C) 60 and (D) 180 minutes after infestation.** Click here for file

Additional file 4 **RNA quality analysis in agarose electrophoresis. (A) Soybean RNA samples collected from different organs at different developmental stages; (B) RNA samples collected from leaves attacked by *A. gemmatalis*, and (C) RNA samples extracted from *M. incognita*-infected roots.** Click here for file

Additional file 5 Reference genes tested for gene expression normalization in soybean under biotic stresses. Click here for file

Additional file 6 Dissociation curves for qPCR products amplified. Click here for file

Additional file 7 **Average value of *Ct *from two biological replicates ± standard deviation (SD) of all 10 genes along all 24 treatments.** Click here for file
