# Supplementary material for: Validation of reference genes aiming accurate normalization of qPCR data in soybean upon nematode parasitism and insect attack
Source: BMC Res Notes. 2013 May 13;6:196. doi: 10.1186/1756-0500-6-196 (PMC3660166; doi:10.1186/1756-0500-6-196)
Supplement: Additional file 1 — Set of samples (organ/treatment) used for gene expression analysis. [file 1756-0500-6-196-S1.pdf]

| Nº | Cultivar | Organ | Developmental stage | Time point | Treatment |
|---|---|---|---|---|---|
| 1 | BRSGO Raíssa | Root | V4 | - | - |
| 2 | BRSGO Raíssa | Stem | V4 | - | - |
| 3 | BRSGO Raíssa | Leaf | V4 | - | - |
| 4 | BRSGO Raíssa | Root | R2 | - | - |
| 5 | BRSGO Raíssa | Stem | R2 | - | - |
| 6 | BRSGO Raíssa | Leaf | R2 | - | - |
| 7 | BRSGO Raíssa | Flower | R2 | - | - |
| 8 | BRSGO Raíssa | Root | R4 | - | - |
| 9 | BRSGO Raíssa | Stem | R4 | - | - |
| 10 | BRSGO Raíssa | Leaf | R4 | - | - |
| 11 | BRSGO Raíssa | Pod | R4 | - | - |
| 12 | Santa Rosa | Root | V2 | 7 DAI | Control – non-inoculated root |
| 13 | Santa Rosa | Root | V4 | 14 DAI | Control – non-inoculated root |
| 14 | Santa Rosa | Root | V5 | 21 DAI | Control – non-inoculated root |
| 15 | Santa Rosa | Root | V6 | 28 DAI | Control – non-inoculated root |
| 16 | Santa Rosa | Root | V2 | 7 DAI | *M. incognita* -inoculated root |
| 17 | Santa Rosa | Root | V4 | 14 DAI | *M. incognita* -inoculated root |
| 18 | Santa Rosa | Root | V5 | 21 DAI | *M. incognita* -inoculated root |
| 19 | Santa Rosa | Root | V6 | 28 DAI | *M. incognita* -inoculated root |
| 20 | BRSGO Raíssa | Leaf | V4 | 0 min | Control – uninfested leaf |
| 21 | BRSGO Raíssa | Leaf | V4 | 15 min | *A. gemmatalis* -infested leaf |
| 22 | BRSGO Raíssa | Leaf | V4 | 30 min | *A. gemmatalis* -infested leaf |
| 23 | BRSGO Raíssa | Leaf | V4 | 60 min | *A. gemmatalis* -infested leaf |
| 24 | BRSGO Raíssa | Leaf | V4 | 180 min | *A. gemmatalis* -infested leaf |
