# Supplementary figures and images for: Validation of reference genes aiming accurate normalization of qPCR data in soybean upon nematode parasitism and insect attack
Source: BMC Res Notes. 2013 May 13;6:196. doi: 10.1186/1756-0500-6-196 (PMC3660166; doi:10.1186/1756-0500-6-196)

## Slide 1
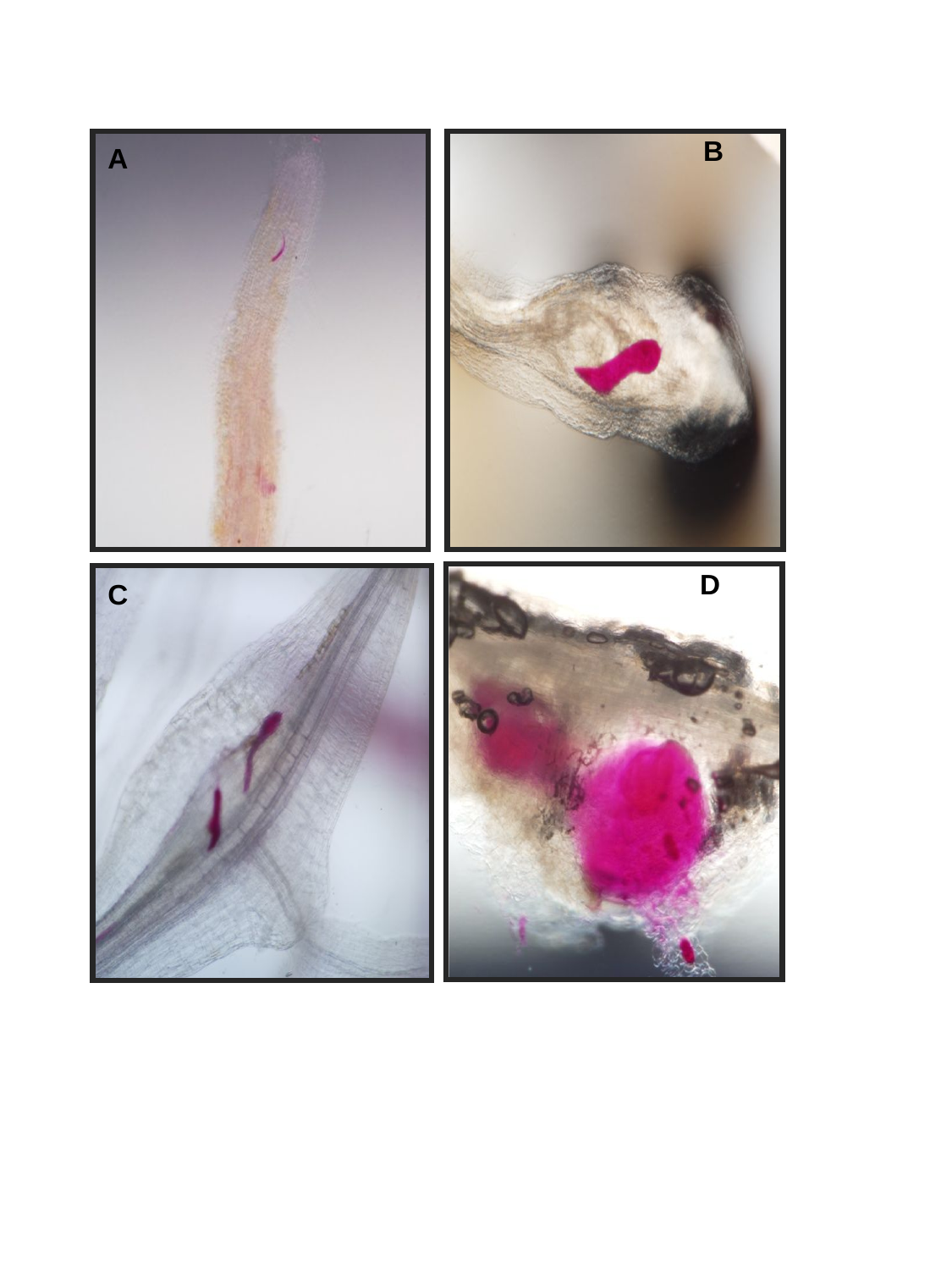

B
A
D
C

Supplement: Additional file 2 — Progress of soybean root infection with M. incognita revealed by acid fuchsin staining. (A) 7 DAI, second-stage juvenile (J2) during penetration and migration into root; (B) 14 DAI, gall formation by J2-J3 in the vascular cylinder; (C) 21 DAI, root knot completely developed; (D) 28 DAI, adult female during egg posture and egg mass. [file 1756-0500-6-196-S2.ppt]

## Slide 1
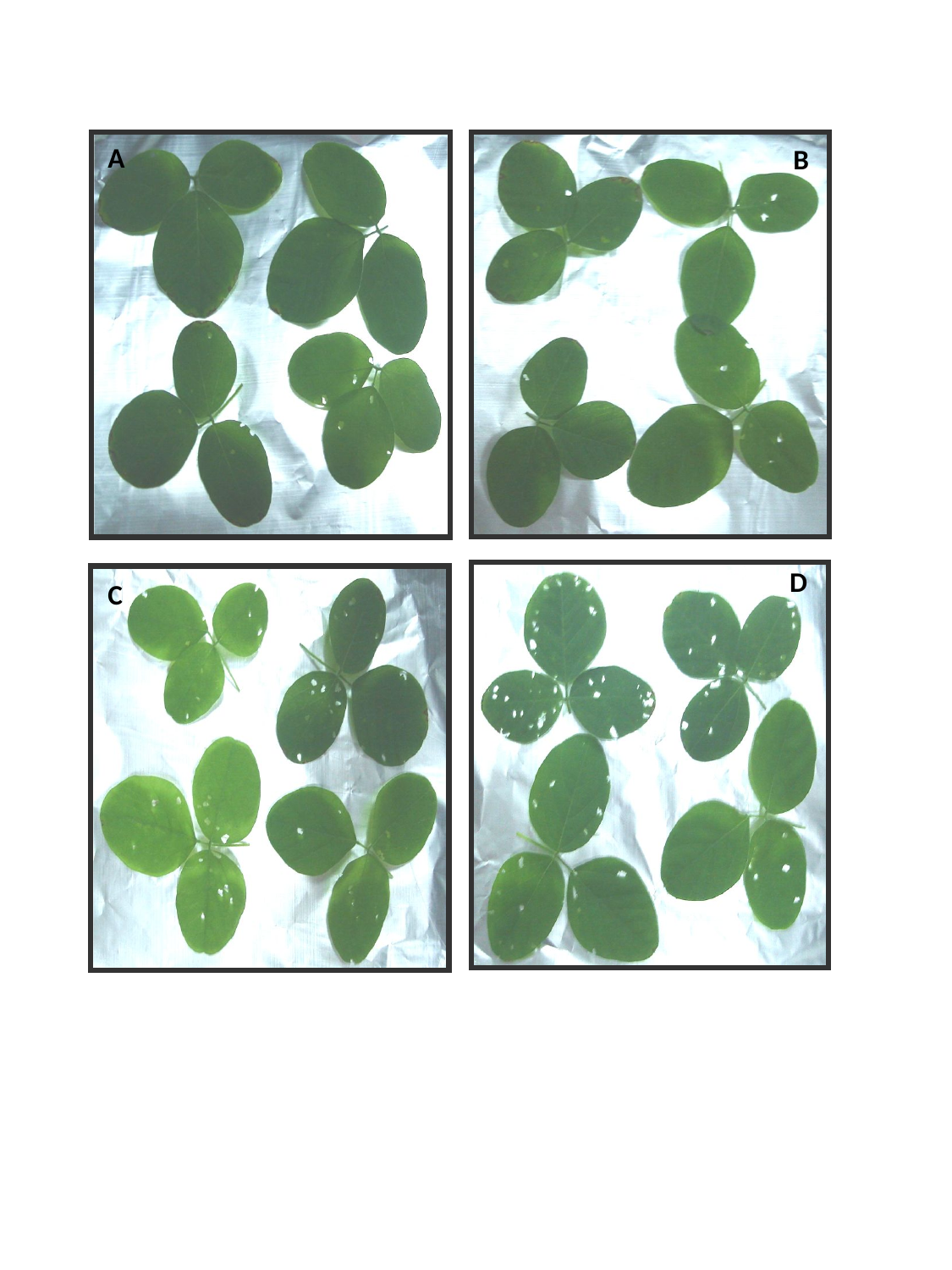

A
B
D
C

Supplement: Additional file 3 — Progress of soybean leaf infestation with A. gemmatalis. Twenty-five larvae of fourth instar of A. gemmatalis were transferred to each soybean trifolium. Leaves were collected at (A) 15, (B) 30, (C) 60 and (D) 180 minutes after infestation. [file 1756-0500-6-196-S3.pptx]

## Slide 1
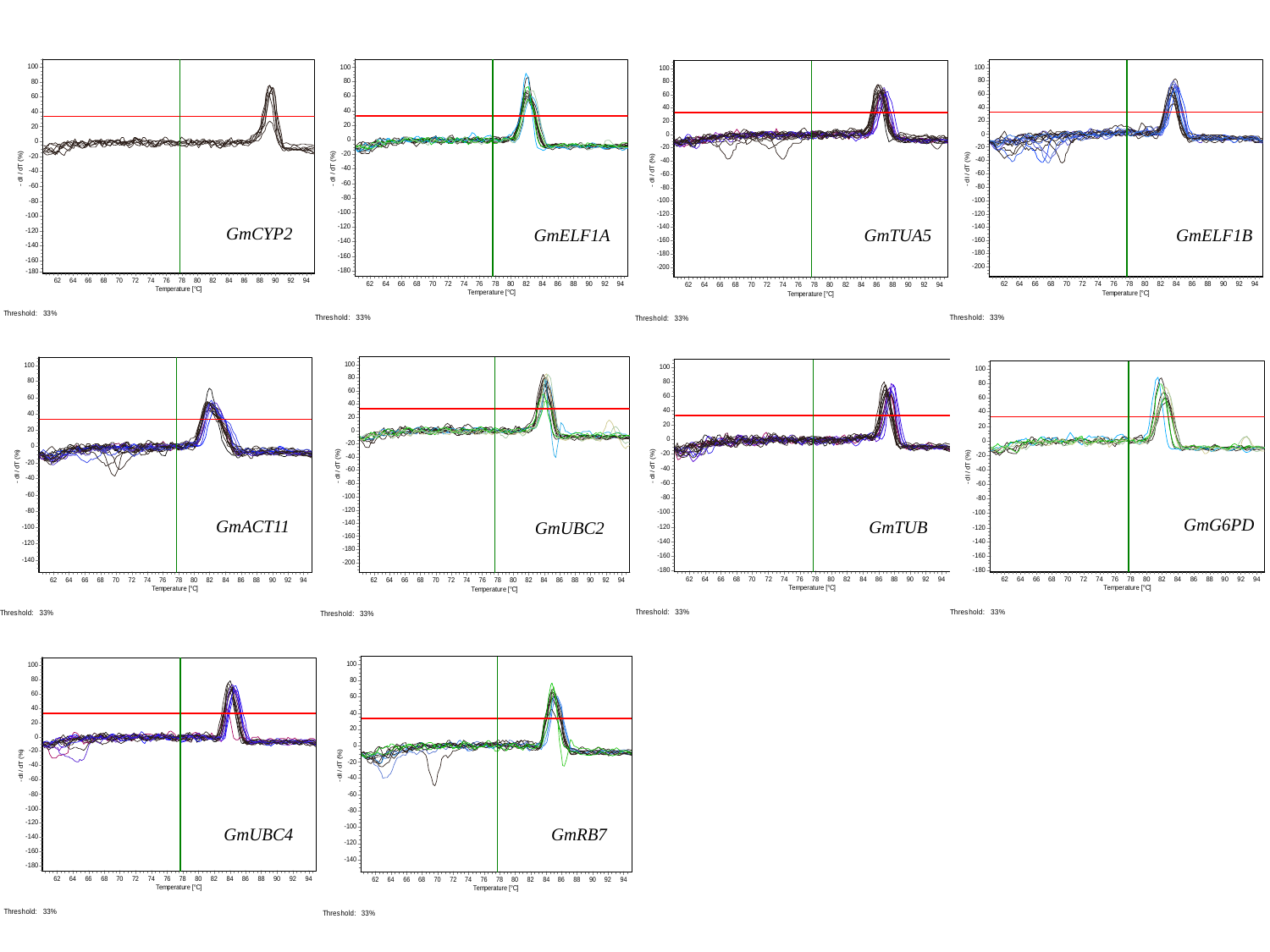

GmELF1A
GmELF1B
GmTUA5
GmCYP2
GmUBC2
GmACT11
GmTUB
GmG6PD
GmRB7
GmUBC4

Supplement: Additional file 6 — Dissociation curves for qPCR products amplified. [file 1756-0500-6-196-S6.ppt]
