# Supplementary material for: Validation of reference genes aiming accurate normalization of qPCR data in soybean upon nematode parasitism and insect attack
Source: BMC Res Notes. 2013 May 13;6:196. doi: 10.1186/1756-0500-6-196 (PMC3660166; doi:10.1186/1756-0500-6-196)
Supplement: Additional file 4 — RNA quality analysis in agarose electrophoresis. (A) Soybean RNA samples collected from different organs at different developmental stages; (B) RNA samples collected from leaves attacked by A. gemmatalis, and (C) RNA samples extracted from M. incognita-infected roots. [file 1756-0500-6-196-S4.ppt]

## Slide 1
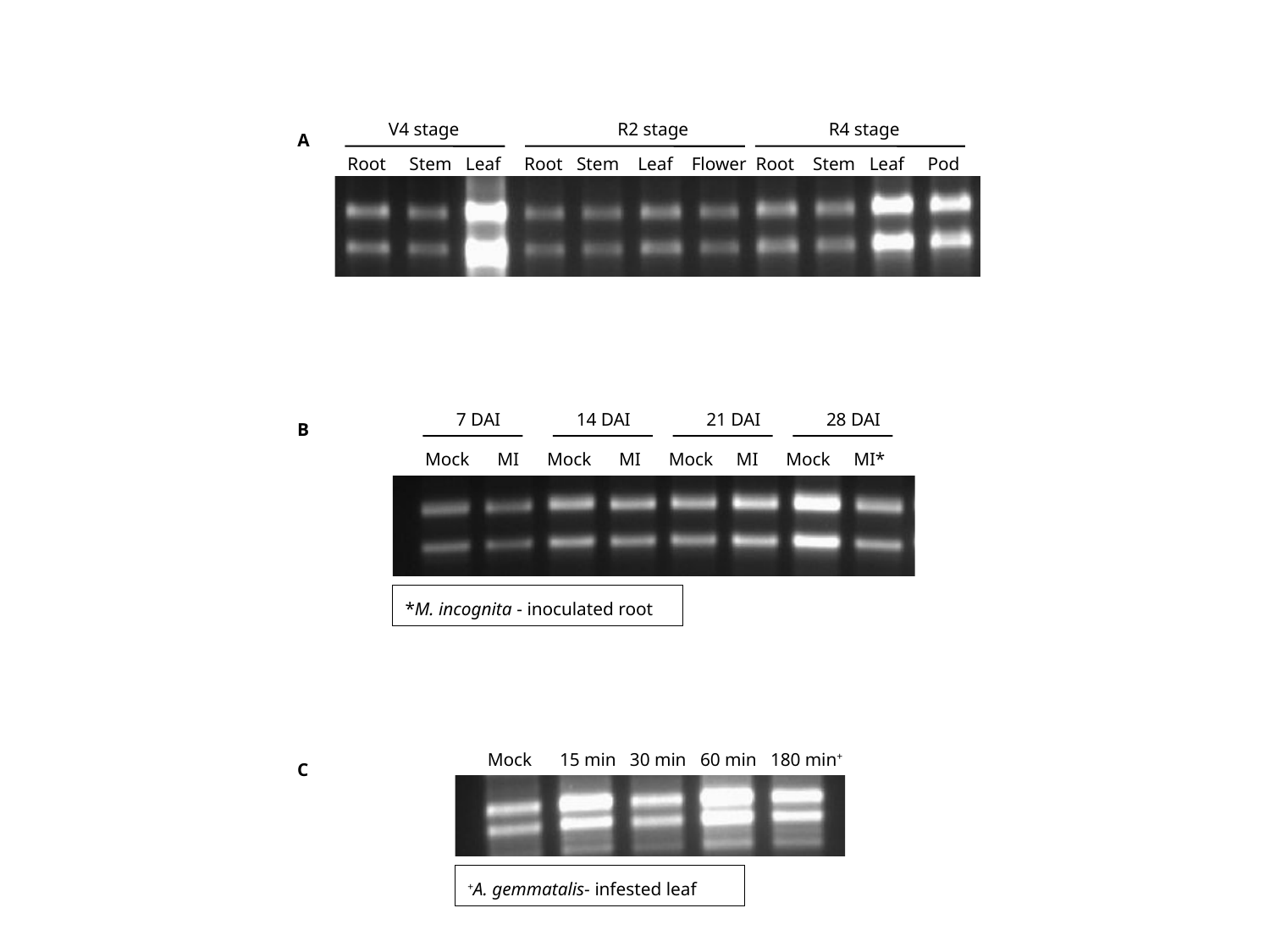

V4 stage
R2 stage
R4 stage
Root Stem Leaf Root Stem Leaf Flower Root Stem Leaf Pod
A
7 DAI
14 DAI
21 DAI
28 DAI
Mock MI Mock MI Mock MI Mock MI*
*M. incognita - inoculated root
B
Mock 15 min 30 min 60 min 180 min+
+A. gemmatalis- infested leaf
C
