# Supplementary material for: Validation of reference genes aiming accurate normalization of qPCR data in soybean upon nematode parasitism and insect attack
Source: BMC Res Notes. 2013 May 13;6:196. doi: 10.1186/1756-0500-6-196 (PMC3660166; doi:10.1186/1756-0500-6-196)
Supplement: Additional file 5 — Reference genes tested for gene expression normalization in soybean under biotic stresses. [file 1756-0500-6-196-S5.pdf]

Gene symbol	*Gene locus*	NCBI Accession number	Gene name	Function
GmCYP2	*Glyma12g02790.1*	CF806591	Cyclophilin 2	Protein folding
GmELF1A	*Glyma05g24110.1*	BT093688.1	Translation elongation factor 1-alpha	Translational elongation
GmTUA5	*Glyma05g29000.1*	CA801144	Tubulin alpha-5	Structural constituent of cytoskeletal
GmELF1B	*Glyma14g04350*	AK286947.1	Translation elongation factor 1-beta	Translational elongation
GmACT11	*Glyma18g52780.1*	BW652479	Actin 11	Structural constituent of cytoskeletal
GmUBC2	*Glyma18g16160*	AK244261.1	Ubiquitin-conjugating enzyme E2 (family UBC2)	Enzyme involved in DNA repair and Endocytosis processes
GmTUB	*Glyma04g09350.1*	AC235288	Beta-tubulin	Structural constituent of cytoskeletal
GmG6PD	*Glyma19g24250*	AK244997.1	Glucose-6-phosphate dehydrogenase	Glycolysis pathway enzyme
GmUBC4	*Glyma18g44850*	AF532622.1	Ubiquitin-conjugating enzyme E2 (family UBC4)	Enzyme involved in abnormal and short-lived proteins degradation
GmRB7	*Glyma11g03690.1*	AI494844.1	Aquaporin	Water channel Integral cell membrane protein
