# Supplementary material for: Validation of reference genes aiming accurate normalization of qPCR data in soybean upon nematode parasitism and insect attack
Source: BMC Res Notes. 2013 May 13;6:196. doi: 10.1186/1756-0500-6-196 (PMC3660166; doi:10.1186/1756-0500-6-196)
Supplement: Additional file 7 — Average value of Ct from two biological replicates ± standard deviation (SD) of all 10 genes along all 24 treatments. [file 1756-0500-6-196-S7.pdf]

V4	*GmCYP2*	*GmELF1A*	*GmTUA5*	*GmELF1B*	*GmACT11*
			***Ct* ± SD**		
Leaf	19.39 ± 0.631	20.30 ± 0.348	20.27 ± 0.498	24.58 ± 0.368	22.34 ± 0.165
Stem	19.066 ± 0.659	19.76 ± 0.649	19.9 ± 0.091	22.63 ± 0.347	20.3 ± 0.846
Root	20.985 ± 1.554	22.51 ± 2.624	22.61 ± 2.466	23.60 ± 1.158	23.61 ± 0.846
**R2**					
Leaf	20.610 ± 0.344	20.61 ± 0.696	21.42 ± 1.063	24.96 ± 0.704	22.78 ± 0.433
Stem	20.152 ± 0.079	20.01 ± 0.267	19.71 ± 0.665	22.81 ± 0.765	20.73 ± 0.342
Root	23.260 ± 0.839	24.29 ± 1.470	25.27 ± 1.393	26.17 ± 1.842	24.83 ± 1.794
Flower	18.824 ± 0.285	18.92 ± 0.499	21.03 ± 2.854	22.61 ± 0.419	20.28 ± 0.224
**R4**					
Leaf	20.464 ± 0.149	21.12 ± 0.311	21.63 ± 0.346	24.65 ± 0.453	22.42 ± 0.370
Stem	20.107 ± 0.721	20.76 ± 0.859	21.68 ± 1.097	22.82 0.378	22.02 ± 0.386
Root	22.300 ± 0.375	23.13 ± 0.978	24.44 ± 1.745	24.16 ± 0.289	23.31 ± 1.303
Pod	19.875 ± 0.443	19.56 ± 0.697	20.82 ± 0.422	23.02 ± 0.468	21.13 ± 0.964
**Efficiency ± SD / CV%**	0.949 ± 0.031 / 6.338	1.157 ± 0.038 / 10.469	1.148 ± 0.028 / 9.202	1.034 ± 0.049 / 9.364	1.118 ± 0.037 / 10.61

V4	*GmUBC2*	*GmTUB*	*GmG6PD*	*GmUBC4*	*GmRB7*
			***Ct* ± SD**		
Leaf	23.78 ± 0.619	24.77 ± 0.494	26.19 ± 0.743	23.06 ± 1.032	20.51 ± 2.140
Stem	22.33 ± 1.595	21.71 ± 0.399	21.95 ± 0.510	22.53 ± 0.286	19.15 ± 1.291
Root	25.65 ± 1.673	27.1 ± 4.981	23.26 ± 1.919	24.64 ± 1.609	19.79 ± 2.720
**R2**					
Leaf	23.99 ± 0.333	28.56 ± 2.348	24.92 ± 0.294	23.78 ± 0.388	24.70 ± 0.686
Stem	23.58 ± 0.710	23.62 ± 3.643	21.99 ± 0.366	23.33 ± 0.232	20.36 ± 0.999
Root	27.015 ± 0.820	28.9 ± 1.285	25.87 ± 2.438	26.48 ± 1.058	22.013 ± 1.424
Flower	23.24 ± 0.717	21.9 ± 0.69	22.49 ± 0.675	23.49 ± 3.000	24.06 ± 1.000
**R4**					
Leaf	24.04 ± 0.357	27.42 ± 0.488	25.52 ± 0.618	23.06 ± 0.237	24.76 ± 1.122
Stem	23.7 ± 0.831	27.89 ± 3.428	22.16 ± 0.434	22.31 ± 0.416	22.25 ± 0.228
Root	25.03 ± 0.121	27.9 ± 1.893	23.09 ± 0.554	24.24 ± 0.769	21.47 ± 1.589
Pod	24.63 ± 0.500	23.44 ± 1.789	24.07 ± 0.806	25.32 ± 4.053	22.94 ± 1.875
**Efficiency ± SD / CV%**	1.076 ± 0.045 / 8.360	1.031 ± 0.032 / 14.575	1.107 ± 0.033 / 11.655	1.091 ± 0.036 / 10.613	0.976 ± 0.053 / 13.537

**Control - uninoculated**	*GmCYP2*	*GmELF1A*	*GmTUA5*	*GmELF1B*	*GmACT11*
			***Ct* ± SD**		
Root (7 d.a.i)	23.92 ± 1.981	23.92 ± 1.252	24.85 ± 1.139	25.07 ± 0.313	23.67 ± 1.581
Root (14 d.a.i)	23.57 ± 1.194	24.62 ± 0.890	24.68 ± 0.660	25.83 ± 1.837	25.35 ± 2.908
Root (21 d.a.i)	23.14 ± 1.348	23.97 ± 0.876	24.95 ± 1.049	25.86 ± 1.316	24.39 ± 1.922
Root (28 d.a.i)	23.85 ± 0.971	24.27 ± 0.727	25.01 ± 0.701	25.25 ± 0.783	24.3 ± 1.609
***M. incognita*-inoculated root**					
Root (7 d.a.i)	24.03 ± 1.836	23.92 ± 0.614	24.73 ± 0.575	25.1 ± 1.847	25.1 ± 3.235
Root (14 d.a.i)	22.38 ± 1.202	23.21 ± 0.823	23.78 ± 0.917	25.98 ± 3.283	23.76 ± 0.910
Root (21 d.a.i)	21.29 ± 0.450	22.4 ± 0.706	23.7 ± 0.399	23.78 ± 0.520	23.22 ± 0.930
Root (28 d.a.i)	21.82 ± 0.346	24.34 ± 1.787	24.52 ± 1.102	24.31 ± 0.581	23.88 ± 1.333
**Efficiency ± SD / CV%**	1.016 ± 0.018 / 12.376	0.892 ± 0.034 / 12.079	0.885 ± 0.025 / 16.414	0.816 ± 0.029 / 15.230	0.965 ± 0.030 / 16.890

**Control - uninoculated**	*GmUBC2*	*GmTUB*	*GmG6PD*	*GmUBC4*	*GmRB7*
			***Ct* ± SD**		
Root (7 d.a.i)	25.780	28.28 ± 1.536	23.030	26.9 ± 3.369	21.13 ± 0.486
Root (14 d.a.i)	23.660	29.46 ± 0.577	23.150	25.86 ± 2.162	21.54 ± 1.962
Root (21 d.a.i)	25.140	28.05 ± 1.009	22.850	26.53 ± 3.300	22.32 ± 5.245
Root (28 d.a.i)	25.920	29.03 ± 0.757	24.900	25.25 ± 1.678	20.87 ± 0.342
***M. incognita*-inoculated root**					
Root (7 d.a.i)	24.340	28.21 ± 1.349	22.14 ±	26.24 ± 4.309	24.7 ± 7.865
Root (14 d.a.i)	25.860	27.95 ± 1.540	23.13 ±	24.33 ± 1.460	19.88 ± 0.719
Root (21 d.a.i)	25.390	27.04 ± 0.730	23.21 ±	23.19 ± 0.464	18.82 ± 5.614
Root (28 d.a.i)	26.030	28.6 ± 1.419	23.87 ±	24.14 ± 1.271	20.54 ± 1.713
**Efficiency ± SD / CV%**	1.04 ± 0.03 / 11.56	0.897 ± 0.024 / 13.170	0.940 ± 0.031 / 21.42	0.870 ± 0.033 / 13.733	0.78 ± 0.029 / 16.962

***A. gemmatalis* infested leaf**	*GmCYP2*	*GmELF1A*	*GmTUA5*	*GmELF1B*	*GmACT11*
			***Ct* ± SD**		
Leaf (0h) control	20.02 ± 0.148	21.57 ± 0.611	24.61 ± 1.526	25.17 ± 0.690	22.69 ± 1.531
Leaf (15 min)	20.06 ± 0.363	21.60 ± 0.357	24.16 ± 0.453	25.33 ± 0.517	22.07 ± 0.450
Leaf (30 min)	21.02 ± 0.188	22.46 ± 0.386	24.10 ± 1.239	26.02 ± 0.606	22.44 ± 0.775
Leaf (1 h)	20.62 ± 0.152	22.16 ± 0.247	24.31 ± 0.711	25.34 ± 0.490	22.35 ± 0.066
Leaf (3 h)	20.86 ± 0.423	22.27 ± 0.316	24.95 ± 0.238	25.75 ± 0.488	22.78 ± 0.127
**Efficiency ± SD / CV%**	1.156 ± 0.018 / 9.25	1.128 ± 0.024 / 8.74	1.051 ± 0.028 / 12.327	0.972 ± 0.025 / 11.31	1.088 ± 0.028 / 12.96

***A. gemmatalis* infested leaf**	*GmUBC2*	*GmTUB*	*GmG6PD*	*GmUBC4*	
			***Ct* ± SD**		
Leaf (0h) control	24.64 ± 0.191	27.89 ± 0.124	24.54 ± 1.632	23.18 ± 1.484	
Leaf (15 min)	24.18 ± 0.68	26.81 ± 0.593	25.11 ± 1.151	22.81 ± 0.658	
Leaf (30 min)	24.63 ± 0.203	28.14 ± 0.551	26.01 ± 1.125	22.95 ± 1.334	
Leaf (1 h)	24.65 ± 0212	27.91 ± 0.455	25.59 ± 1.067	23.25 ± 1.535	
Leaf (3 h)	24.99 ± 0.309	28.53 ± 1.034	25.45 ± 1.248	23.92 ± 1.256	
**Efficiency ± SD / CV%**	1.075 ± 0.028 / 12.27	1.027 ± 0.020 / 11.58	1.032 ± 0.025 / 12.675	0.943 ± 0.027 / 15.41	
